# Aberrant expression of multiple γ-glutamyltransferases is associated with tumor progression and patient outcome in prostate cancers

**DOI:** 10.3389/fonc.2025.1518636

**Published:** 2025-02-28

**Authors:** Wencong Jiang, Wang Liu, Jiang Zhao, Zhijian Xu, Ming Xi, Xiangwei Wang, Benyi Li

**Affiliations:** ^1^ Department of Urology, Huadu District People’s Hospital, Guangzhou, China; ^2^ Department of Urology, The University of Kansas Medical Center, Kansas City, MO, United States; ^3^ Department of Urology, The Affiliated Hospital of Guangdong Medical University, Zhanjiang, China

**Keywords:** prostate cancer, GGT family genes, castration-resistance, neuroendocrinal progression, DNA methylation

## Abstract

**Introduction:**

The human gamma-glutamyltransferase (GGT) is a membrane-bound extracellular glycoprotein with an enzymatic activity that cleaves gamma-glutamyl peptide bonds in glutathione and other peptides and transfers the gamma-glutamyl moiety to acceptors. It has been shown aberrant expression of GGT proteins in human cancers while their expression profiles in prostate cancers are not reported.

**Methods:**

In this study, we analyzed the expression profiles of all protein-coding GGT genes using the TCGA-PRAD RNA-seq dataset derived from primary prostate cancers. GGT family gene expression profiles were also analyzed using the SU2C/PCF RNAseq dataset derived from aggressive late-stage prostate cancer patients. Androgen modulation of GGT family gene expression was analyzed using multiple NCBI/GEO datasets.

**Results:**

Our results showed that prostate tissues expressed four major isoforms of GGT family genes (GGT1/5/6/7), of which GGT1 expression was upregulated but GGT6/GGT7 expression was downregulated in cancer tissues compared to benign tissues. However, GGT5 expression was increased along with tumor stage progression and associated with worse progression-free survival. GGT6 expression exhibited a superb AUC value in prostate cancer diagnosis and was associated with favorable progression-free survival. GGT1 expression was highly increased but GGT6/GGT7 expression was largely reduced in ERG-fusion-positive cases. In CRPC tumors, GGT6 expression was suppressed in patients with anti-AR therapies, which was reversed when patients were taken off the treatment. This AR-dependent modulation was confirmed in LNCaP cells and LuCaP35 xenograft models. In addition, compared to CRPC-Adeno tumors, treatment-induced NEPC tumors showed a reduced GGT1 but an elevated GGT7 level, which was in line with higher levels of GGT7 in NEPC H660 cells.

**Conclusion:**

Our data suggests that GGT6 is a new AR downstream target but GGT7 is a potential NEPC biomarker.

## Introduction

The human gamma-glutamyltransferase (GGT; EC 2.3.2.2) gene family includes 11 genes, 4 full-length proteins with both heavy and light chains (GGT1/GGT5/GGT6/GGT7), 3 light chain-only proteins (GGTLC1/GGTLC2/GGTLC3), and 4 pseudogenes (GGT2P/GGT3P/GGT4P/GGT8P) (1). The full-length proteins are membrane-bound extracellular enzymes anchored onto the plasma membrane with a short N-terminal transmembrane domain (1). The major physiological function of these GGT enzymes is to break down extracellular glutathione (GSH) that cannot be taken up by most cells. GGT enzymes hydrolyze the γ-glutamyl group on GSH releasing cysteinyl-glycine dipeptide, which will be broken down into cysteine and glycine by cell surface dipeptidases for cellular uptake (2). GGT enzymes also convert leukotriene LTC4 to LTD4 (3, 4) and cleave γ-glutamyl peptide bonds in other peptides (5), resulting in γ-glutamyl moiety transfer to acceptors (6). These functionalities are critical for cellular GSH homeostasis in anti-oxidative defense, inflammatory molecule synthesis, and drug metabolism (6, 7).

GGT genes are ubiquitously expressed in plant and mammalian cells, the most studied isoforms are GGT1 and GGT5 with well-known enzymatic activities (6), while the functional role of GGT6/GGT7 is still unclear (1). GGT1/GGT5 proteins are often expressed by different cell types within the tissue. For example, GGT1 protein is expressed on the apical surface of the renal proximal tubules while GGT5 protein is expressed by the interstitial cells of the kidney. GGT1/GGT5 are moderately expressed on the secretory or absorptive cells in sweat glands, prostate, liver, bile ducts, pancreatic acini, intestinal crypts, and testicular tubules (6). These different patterns of expression are responsible for their distinct substrate specificity. For instance, GGT5 uses the substrates in blood and intercellular fluids, while GGT1 mainly uses the substrates in duct and gland fluids throughout the body (8). Since its abundant expression in liver cells, hepatic toxicity-induced liver cell damage results in the blood release of GGT proteins, which has been used as a biomarker for liver disease (9). Genetic knockout studies in mice showed that GGT1 and GGT5 both enzymes can convert leukotriene C4 to leukotriene D4, but they have (9).

In human prostate cancers, higher GGT1-derived γ-glutamyltransferase activity was recently reported in exosomes isolated from patient blood samples compared to benign prostatic hypertrophy (BPH) individuals (10). GGT1 protein levels in exosomes were significantly higher in aggressive C4-2B cells compared to less aggressive LNCaP cells, suggesting that GGT1 expression is associated with disease progression in prostate cancer. However, it is largely unknown the expression patterns of all GGT family genes in prostate cancers and their correlations with disease progression and patient survival outcomes. In this study, we took advantage of public RNA-seq datasets and analyzed the expression profiles of all GGT family genes in prostate cancers. We also investigated the androgen receptor (AR) modulation of GGT family gene expression in prostate cancer cells. Our results indicate that GGT6 expression is modulated by AR signaling activity and GGT7 expression is highly increased in treatment-induced neuroendocrinal progression.

## Results

### GGT family genes were aberrantly expressed in primary prostate cancers

**Figure 1:**
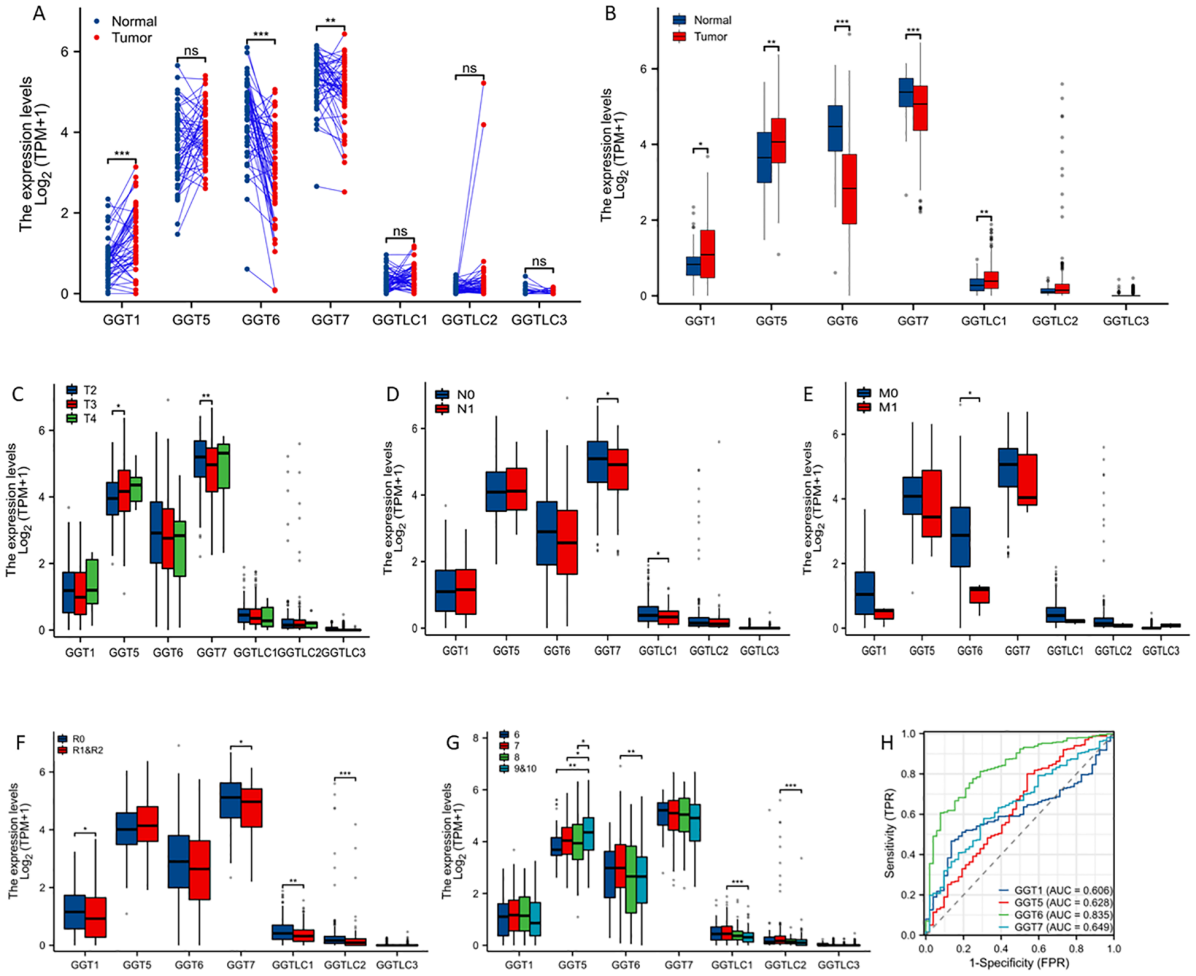
GGT family genes are dysregulated in prostate cancers. Case-matched pair-wise approach **(A)** or group cohort comparison **(B)** were used to compare gene expression levels between benign and cancer tissues using the TCGA-PRAD dataset. Gene expressions were also compared in clinical subgroups, including clinical stage **(C)**, lymph node invasion **(D)**, distal metastasis **(E)**, post-surgery residual tumor **(F)**, and Gleason scores **(G)**. The asterisks indicate a significant difference compared to the control group. *p < 0.05; **p < 0.01, ***p < 0.001. **(H)** ROC analysis was conducted to determine the prediction potentials in distinguishing normal and tumor tissues. ns; no significance.

We used the TCGA-PRAD dataset to examine the expression profiles in primary prostate cancers. Gene expression levels were compared in two comparisons, case-matched pair (52 cases) and group cohort (501 tumors). The predominant isoforms in prostate tissues were GGT5/6/7 genes while the GGT1 gene was expressed at a moderate level. In contrast, the light-chain-only isoform GGTLC1/2/3 genes were expressed at a very low level. Compared to benign tissues, the GGT1 gene was significantly upregulated while GGT6/7 genes were significantly downregulated in case-matched pair comparison (**Figure 1A**). Similar results were also observed in group cohort comparisons for these genes, except that GGT5 and GGTLC1 expression levels were significantly higher in tumors compared to benign tissues (**Figure 1B**). Genetic alterations in these genes were only seen in 1-2.6% of cases with very few mutations (**Supplementary Figure S1**, **Supplementary Table S1**).

We then analyzed the association of GGT gene expression with clinicopathological parameters. GGT5 gene expression was significantly higher in T3 diseases while GGT7 expression was significantly lower in T3 diseases compared to T2 diseases (**Figure 1C**). The expression levels of GGT7 and GGTLC1 genes were significantly reduced in patients with lymph node invasion (**Figure 1D**). Interestingly, GGT6 expression was dramatically reduced in cases with distal metastasis, indicating a potential metastatic suppressor (**Figure 1E**). In cases with post-surgical residual tumors, multiple GGT isoform genes (GGT1/GGT7/GGTLC1/GGTLC2) were expressed at a significantly lower level compared to cases without residue tumors (**Figure 1F**). GGT5 gene expression was significantly correlated with increasing Gleason scores while GGT6 expression was significantly lower in Gleason 9 cases compared to Gleason 6 cases (**Figure 1G**). ROC analysis revealed that GGT6 gene expression exerted a strong factor in distinguishing tumor tissues from benign tissues with an AUC value of 0.835 (**Figure 1H**).

### GGT family genes were distinctly associated with multiple molecular signatures

**Figure 2:**
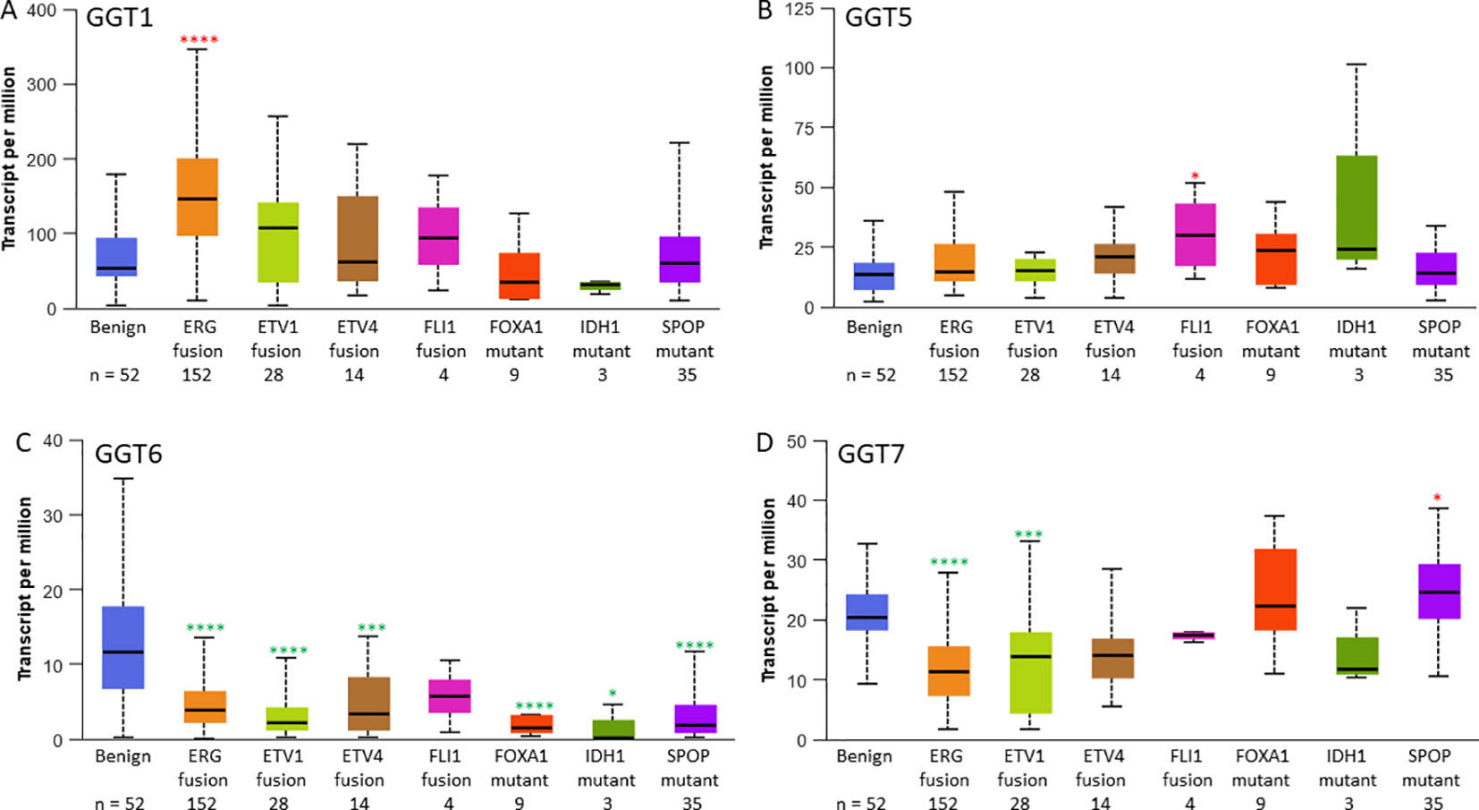
GGT family gene expression levels are changed in subgroups with specific molecular signatures in prostate cancers. Gene expression of GGT1 **(A)**, GGT5 **(B)**, GGT6 **(C)**, and GGT7 **(D)** in the TCGA-PRAD patients. The asterisks indicate a significant difference compared to the benign group. *p < 0.05; ***p < 0.001, ****p < 0.0001.

We then analyzed GGT family gene expression patterns with molecular signatures. GGT1 expression was highly expressed in ERG-fusion positive cases (**Figure 2A**) while GGT5 expression was significantly higher in FLI1-fusion cases (**Figure 2B**). In contrast, GGT6 expression was reduced in almost all cases except FLI1 fusion patients (**Figure 2C**), whereas GGT7 expression was significantly downregulated in cases with either ERG- or ETV1-fusion but significantly upregulated in patients with SPOP mutations (**Figure 2D**).

### GGT5/GGT6/GGT7 expression was associated with disease progression

**Figure 3:**
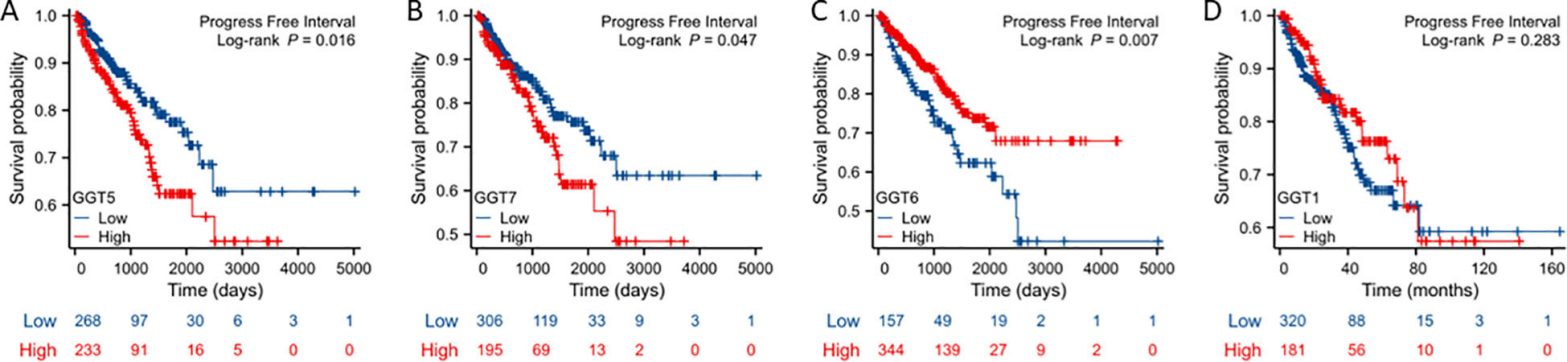
Kaplan-Meier survival analysis was conducted to analyze the association of GGT family genes (**A**: GGT5; **B**: GGT7; **C**: GGT6; **D**: GGT1, as indicated) with patient progression-free interval.

We next analyzed the association of GGT expression with patient survival outcomes, including overall survival, disease-specific survival, and progression-free interval. Kaplan-Meyer curve analysis revealed that GGT5/GGT7 expression was associated with a worse outcome in progression-free intervals (**Figures 3A, B**) while GGT6 expression exhibited a favorite association with progression-free interval (**Figure 3C**). GGT1 expression had no significant association with survival outcomes (**Figure 3D**). Meanwhile, there was no significant association between the expression of all GGT family genes and overall or disease-specific survival outcomes.

### GGT6 expression was reversely correlated with DNA methylation

**Figure 4:**
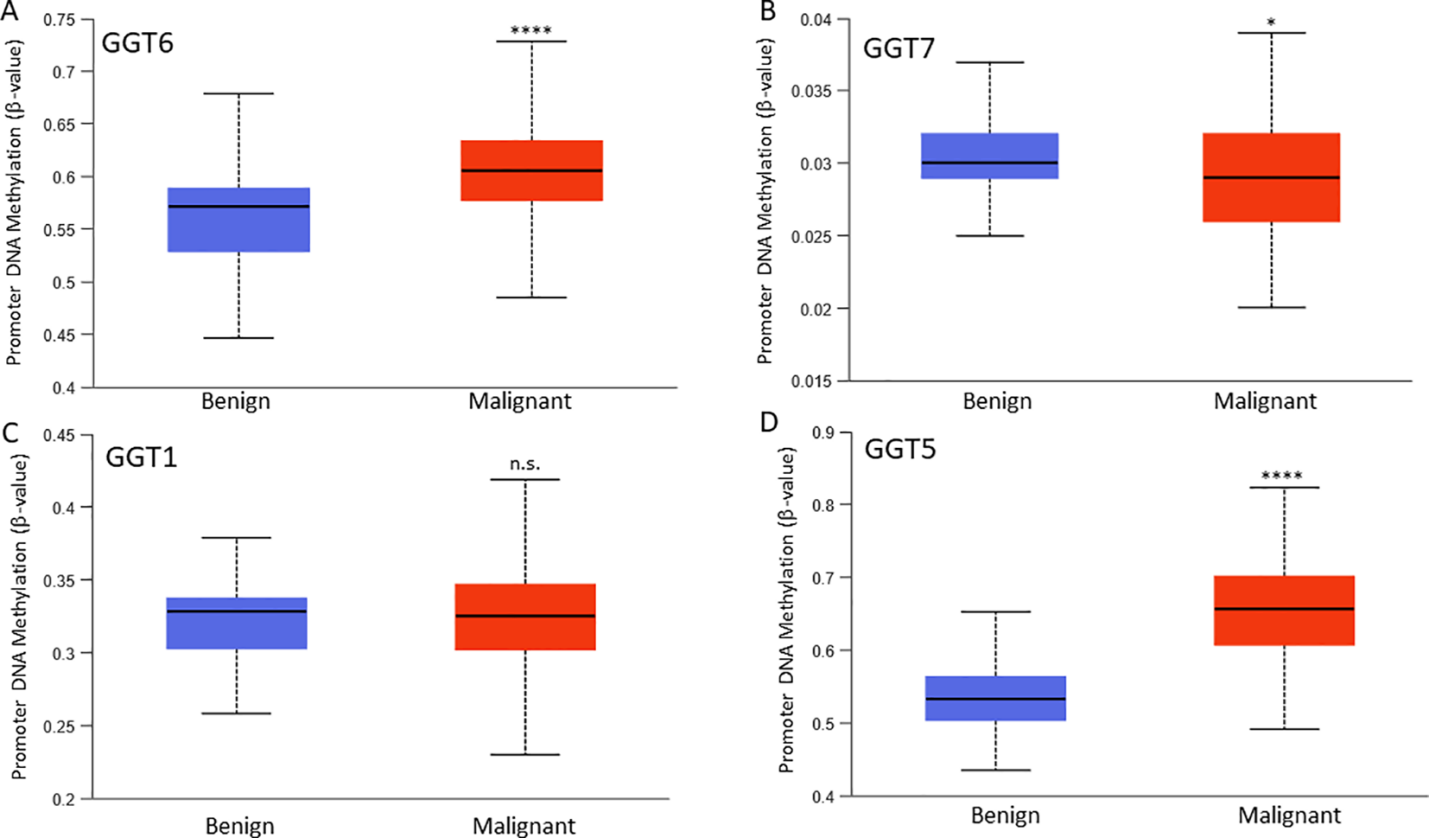
Promoter DNA methylation. DNA methylation at the β-value was compared between benign and cancer tissues for GGT6 **(A)**, GGT7 **(B)**, GGT1 **(C)**, and GGT5 **(D)**. The asterisks indicate a significant difference compared to the benign group. *p < 0.05, ****p < 0.0001. ns; no significance.

We examined the correlation of gene expression levels with promoter DNA methylation levels. Our results showed very strong negative correlations between the expression levels of GGT1/GGT5/GGT6 genes, and their DNA methylation levels in prostate cancer tissues (**Table 1**). While the GGT6 gene exerted the strongest negative correlation (co-efficient > -0.8), GGT7 expression only showed a weak correlation with DNA methylation. We also compared the DNA methylation levels between benign and cancer tissues for each gene (**Figure 4**). Consistent with GGT6 downregulation and the strong negative correlation, the GGT6 promoter showed a significantly higher methylation level in cancer tissue compared to benign tissues (**Figure 4A**). GGT7 methylation level had a moderate increase, which was still in line with a moderate downregulation of GGT7 expression (**Figure 4B**). However, GGT1 methylation had no significant alteration (**Figure 4C**) and GGT5 methylation was significantly elevated in cancer tissues (**Figure 4D**), which is not in line with the upregulation of GGT5 expression in cancer tissues. These data indicate a diversity of regulatory mechanisms for the GGT family genes.

**Table 1:** Correlation of GGT gene expression with DNA Methylation.

Gene	Spearman r	p value	Pearson r	p value	R^2^
GGT1	-0.61	2.21E-51	-0.53	1.30E-37	0.28
GGT5	-0.4	3.70E-20	-0.4	8.78E-21	0.16
GGT6	-0.84	8.60E-133	-0.8	6.34E-112	0.64
GGT7	-0.26	2.27E-09	-0.32	1.39E-13	0.1
GGTLC1	0.02	6.14E-01	0.01	8.26E-01	0
GGTLC2	-0.02	7.11E-01	-0.02	6.79E-01	0

### GGT6 expression was modulated by androgen receptor signaling in CRPC patients

**Figure 5:**
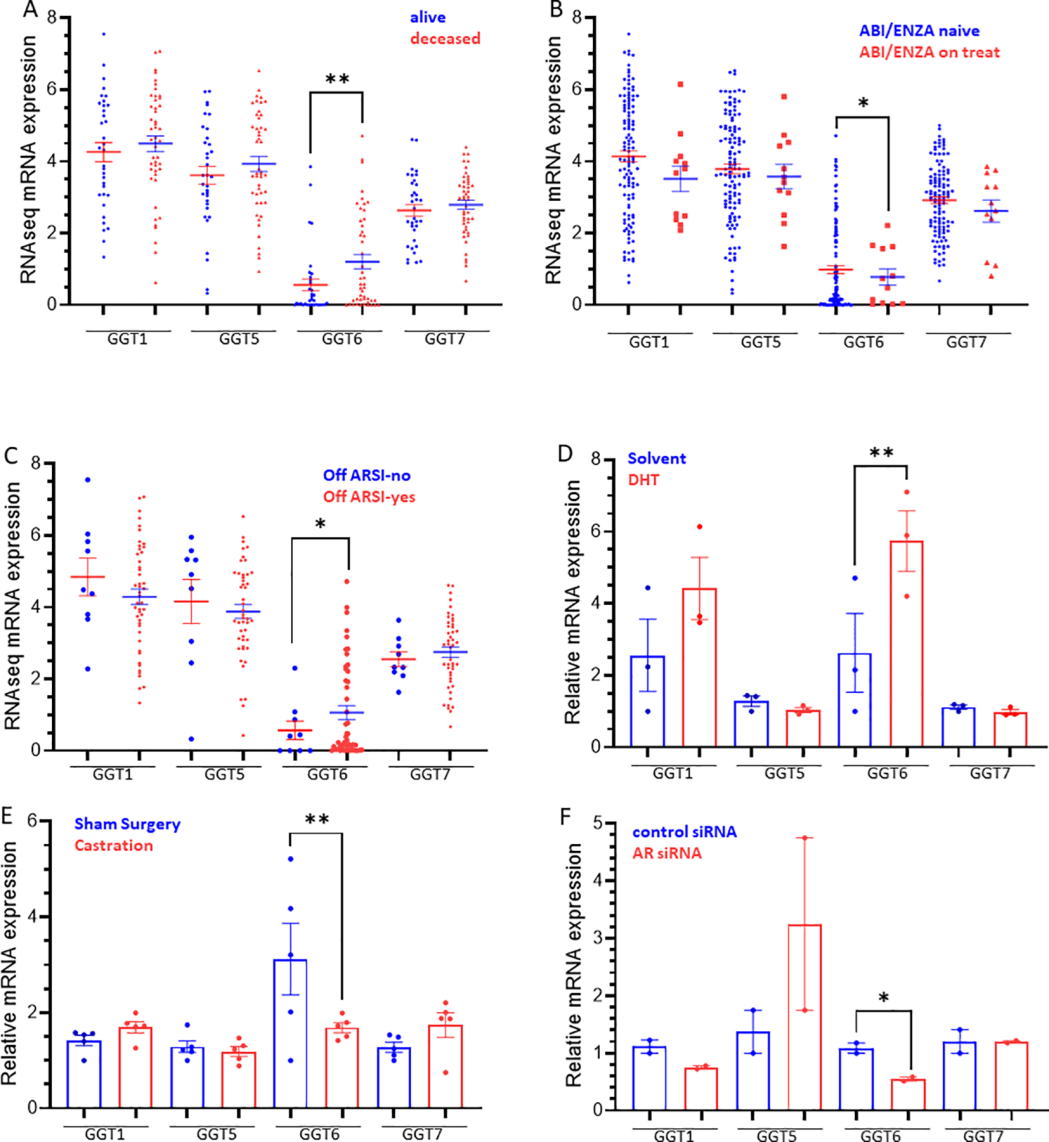
**(A–C)** GGT family gene expression in CRPC patients. Gene expression was compared based on patient survival status **(A)**, anti-AR therapy **(B)**, ARSI status **(C)**, or in response to androgen treatment **(D)**, Castration **(E)**, and AR silencing **(F)**. The asterisk indicates a significant difference between the two groups. *p < 0.05, **p < 0.01.

We analyzed the expression profiles in castration-resistant prostate cancers (CRPC) using the SU2C/PCF RNA-seq dataset. Similar to the TCGA dataset derived from primary prostate cancers, genetic alterations in CRPC tumors were also within 1.6-4% of the GGT family genes (**Supplementary Figure S2**, **Supplementary Table S1**). There was no identical mutation between these two cohorts on each GGT gene. Among these GGT genes, GGT6 was the only one that exerted a significant upregulation in deceased patients compared to alive ones that were alive (**Figure 5A**), which contrasted with its expression profile in primary cancers with a downregulated expression in cancer tissues (**Figure 1A**). Interestingly, GGT6 expression was suppressed in patients receiving Abiraterone or Enzalutamide treatment (**Figure 5B**), which was reversed when patients were off-treatment (**Figure 5C**). This data strongly indicates an AR-dependent modulation of GGT6 expression.

We then analyzed GGT expression in prostate cancer cells after androgen treatment. LNCaP cells were treated with DHT for 4 h significantly increased GGT6 expression and a moderate increase of GGT1 expression without statistical significance (**Figure 5D**). Consistently, castration of the animals bearing the LuCaP35 xenograft tumors significantly reduced GGT6 expression (**Figure 5E**). AR gene silencing in LNCaP cells resulted in a significant reduction of GGT6 expression (**Figure 5F**). These data demonstrated that AR activity is involved in GGT6 expression in prostate cancers.

### GGT7 expression was upregulated in NEPC tumors

**Figure 6:**
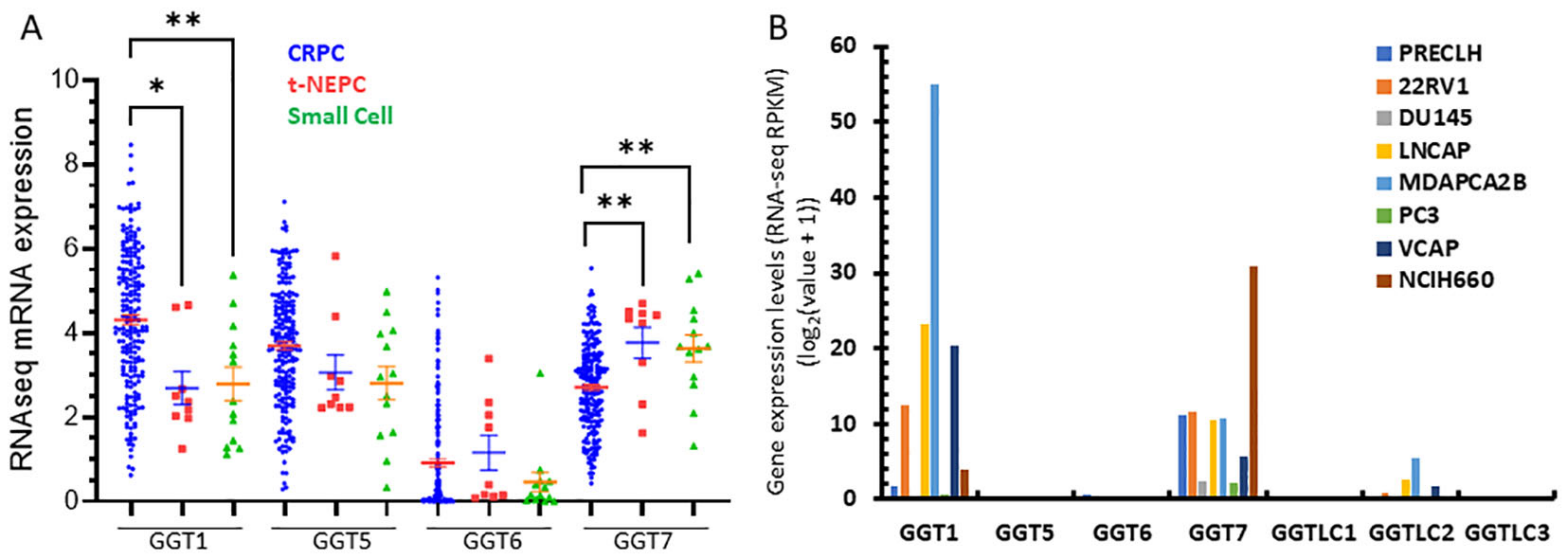
GGT1/GGT7 expression was altered in t-NEPC cancers. Gene expression was compared among different patient groups **(A)** and prostate cell lines **(B)** as indicated. The asterisk indicates a significant difference compared to the CRPC group. Student’s t-test, *p < 0.05, **p < 0.01.

Finally, we analyzed the correlation of GGT family expression profiles with AR activity score or neuroendocrinal progression score (NEPC score) in CRPC tumors. GGT1 expression was positively correlated with the AR score but negatively correlated with the NEPC score, while GGT5/GGT6 genes only showed a very weak correlation with the AR score but not with the NEPC score (**Table 2**). In contrast, GGT7 expression was negatively correlated with AR score but positively correlated with NEPC score in CRPC patients (**Table 2**). Indeed, GGT1 expression levels were significantly lower in tumors with treatment-induced NEPC (t-NEPC) features and small cell carcinomas (SCC) compared to CRPC-adenocarcinomas (CRPC-Adeno). On the other hand, GGT7 expression levels were significantly higher in t-NEPC and SCC tissues compared to CRPC-Adeno tumors (**Figure 6A**). In supporting these data, the NEPC cell line NCI-H660 expressed the highest level of GGT7 expression among all prostate cancer cell lines (**Figure 6B**). These results indicate that GGT7 expression is upregulated in NEPC tumors, representing a potential novel biomarker for neuroendocrinal progression.

**Table 2:** Correlation of GGT gene expression with AR/NEPC scores.

AR score	Spearman r	p value	Pearson r	p value	R^2^
GGT1	0.22	2.88E-04	**0.33**	**6.36E-08**	**0.11**
GGT5	-0.27	7.03E-06	-0.14	0.0256	0.02
GGT6	-0.19	1.57E-03	-0.11	n.s.	0.01
GGT7	-0.32	1.68E-07	**-0.35**	**5.74E-09**	**0.12**
NEPC score					
GGT1	**-0.41**	**3.75E-12**	**-0.43**	**1.17E-11**	**0.16**
GGT5	-0.07	n.s.	-0.13	0.033	0.02
GGT6	-0.04	n.s.	-0.04	n.s.	0
GGT7	0.27	6.22E-06	**0.35**	**6.23E-09**	**0.12**

ns; no significance.

Bold values indicates a strong correlation.

## Discussion

Despite prostate cancer as the second leading cause of cancer deaths in men worldwide, diagnosis and prognosis methods remain limited without curative treatments (11, 12). Most prostate cancer patients with distal metastasis receive androgen deprivation therapy followed by anti-AR treatment once the tumor relapses at the castration-resistant stage (CRPC) (12, 13). About 20-25% of CRPC patients progress into the neuroendocrinal stage (NEPC) after AR antagonist therapy (12). Currently, there is no adequate treatment for NEPC patients other than traditional chemotherapy. Therefore, it is urgent to develop useful biomarkers for monitoring NEPC progression and treatment selection.

In this study, we surveyed the GGT gene family for their expression profiles in primary prostate cancers and treatment-resistant prostate cancers. RNA-seq data from the TCGA-PRAD dataset were derived from primary prostate cancers and the SU2C/PCF dataset was derived from CRPC patients (14). Our results showed that GGT1/5/6/7 genes are the predominant isoforms while GGTLC1/2/3 is expressed at a very low level in prostate tissues, although there are some outliers with higher levels of GGTLC2 in primary prostate cancers. Further analysis revealed that these outliers with higher GGTLC2 expression were Gleason score 7 non-metastatic cases at T2-T3 stages. Compared to case-matched benign tissues, most cases of cancer tissues expressed higher GGT1/GGT5 genes but lower GGT6/GGT7 genes, of which GGT6 expression showed a dramatic reduction in cancer tissues. ROC analysis indicated GGT6 as a reliable marker for prostate cancer diagnosis (AUC = 0.835). Unlike GGT1/GGT5 with confirmed enzymatic activity towards GSH and LTC4, the enzymatic activity of GGT6/GGT7 and GGTLC1/2/3 proteins is not confirmed yet (1, 6). Therefore, the clinical significance and potential mechanistic role in cancer progression of their alterations at the mRNA level remain further investigated.

The GGT6 gene is the least homolog away from GGT1 compared to GGT5/GGT7 (1). Its expression pattern at the protein level is unknown, however, GGT6 mRNA level was recently found to correlate with disease progression and patient outcomes in oral squamous cell carcinoma (15), papillary renal cell carcinoma (16), glioblastoma (17), hepatocellular carcinoma (18), and head-neck squamous cell carcinoma (19). In this study, we were the first to report that GGT6 expression was sharply downregulated in primary prostate cancers, which might be due to enhanced promoter DNA methylation. In addition, GGT6 expression was suppressed during anti-AR treatment, which was reversed after finishing the treatment in CRPC patients. Further analysis in cell culture and xenograft models demonstrated that GGT6 expression was modulated at the transcription level by AR signal activity in prostate cancer. CRPC patients who died early expressed higher GGT6 mRNA levels compared to alive patients. The clinical and biochemical significance of this alteration is under further investigation.

The GGT7 gene is the newest member of the GGT family and is expressed at the highest level in human brain tissue followed by the thyroid gland (1, 20). GGT7 expression is downregulated in glioblastoma (20) but upregulated in liver cancers (18). In this study, we found that GGT7 was slightly downregulated in primary prostate cancer tissues with lymph node invasion and post-surgery residue tumors. However, GGT7 expression was higher in patients with SPOP mutation than others, and GGT7^high^ patients had worse progress-free survival. In CRPC patients, GGT7 expression was negatively correlated with AR score but positively with NEPC score. However, androgen treatment or anti-AR therapy had no effect on GGT7 expression at the mRNA level. Interestingly, GGT7 expression was significantly higher in CRPC patients with neuroendocrinal features and small-cell carcinomas. Meanwhile, the only NEPC cell line H660 is expressing a very high level of GGT7 mRNA. These results strongly suggest that GGT7 might play an important role in NEPC progression or serve as a biomarker for NEPC diagnosis.

In conclusion, GGT1/GGT5 expression was upregulated but GGT6/GGT7 expression was downregulated in primary prostate cancers. Due to a sharp reduction, GGT6 expression exhibited a superb indication for prostate cancer diagnosis. GGT6 expression in CRPC patients was modulated by AR signal activity but GGT7 expression is a potential NEPC biomarker.

## Materials and methods

### Gene expression profiles and genetic alteration in prostate cancers and cell lines

The Cancer Genome Atlas program (TCGA-PRAD) RNAseq dataset was used to survey the gene expression profiles at the mRNA level in primary prostate cancer, as described in our recent publications (21–29). Data statistical analysis and visualization were conducted on the XIANTAO online platform (https://www.xiantaozi.com/). Gene expression levels were compared using two approaches, case-matched pair comparison (52 cases) and group cohort comparison (500 patient cases) with 52 benign samples. Patients were also stratified into subgroups based on multiple clinicopathological parameters to analyze gene expression differences. Comparison of gene expression levels in subgroups stratified by molecular signatures (distinct gene fusion and common mutations) was conducted using the TCGA-PRAD dataset on the UALCAN platform (https://ualcan.path.uab.edu/). Gene expression data in prostate cell lines were downloaded from the Cancer Cell Line Encyclopedia dataset (30, 31) on the cBioportal platform.

Genetic alterations of the GGT family genes were analyzed using the whole genomic sequencing dataset derived from the TCGA-Firehose Legacy program which has 501 tumor samples from primary prostate cancers and the SU2C/PCF program which has 444 CRPC patient samples. Structural deletion, amplification, and mutation data were downloaded and visualized on the cBioportal platform (https://www.cbioportal.org/).

### Correlation analysis of gene expression with promoter DNA methylation

The correlations between the gene expression level of GGT family genes and promoter methylation were analyzed using the TCGA-PRAD sequencing dataset on the cBioportal platform. We used two statistical approaches, Spearman and Pearson coefficients, for the correlation analysis. In addition, promoter DNA methylation level (β-value) was analyzed on the UALCAN platform, as described (32, 33). Different β-value cut-off was considered to indicate hyper-methylation [β-value: 0.7 - 0.5] or hypo-methylation [β-value: 0.3 - 0.25].

### Patient survival outcome assessment

Three survival outcomes, including overall survival, disease-specific survival, and progression-free interval, were assessed using the Kaplan-Meier curve approach with the TCGA-PRAD dataset on the XIANTAO platform. Patients were stratified using the minimum *p*-value cut-off approach (34). The results were visualized on the XIANTAO platform with the survminer package and ggplot2 package of the R package (version 4.2.1).

### Gene expression analysis in CRPC patients

Gene expression at the mRNA levels in CRPC patients was analyzed using the SU2C/PCF RNA-seq dataset downloaded from the cBioportal platform. Patients were divided into different subgroups based on survival status and treatment history with anti-AR therapy for comparison. Correlations between gene expression levels and AR score or NEPC score were also assessed in both categories, Spearman and Pearson coefficients.

### Androgen modulation of gene expression in prostate cancer cell line and xenograft models

Human prostate cancer LNCaP cells were seeded in RPMI1640 media. After serum starvation overnight LNCaP cells were treated with 5-α-dihydrotestosterone (DHT, 0.1 µM) for 4 h in media supplied with charcoal-stripped fetal bovine serum (cFBS) (35). Total cellular RNAs were extracted using a QIAGEN RNeasy kit (Valencia, CA) for GeneChip assay (human U133 plus 2.0). The results were downloaded from NCBI GEO profile GDS3111.

To evaluate the effect of castration on gene expression, human prostate cancer LuCaP35 xenograft models were established subcutaneously in NOD/SCID mice (36). After castration or sham operation, xenograft tumors were harvested for RNA extraction using the QIAGEN RNeasy Mini Kit (Valencia, CA) for GeneChip assays using the Affymetrix human genome U133 Plus 2.0 array. The results were downloaded from the NCBI GEO profile GDS4120.

To confirm the involvement in androgen modulation of gene expression, AR expression was silenced in LNCaP cells with a small-hairpin RNA lentivirus or a nontargeting control siRNA (37). After three days, total cellular RNAs were prepared using the QIAGEN RNeasy Mini kit (Valencia, CA) for gene chip assay using the Affymetrix human U133 Plus 2.0 microarrays. The results were downloaded from NCBI GEO profile GDS4113.

### Statistical analysis

Gene expression at the mRNA levels was used as Log_2_ [TPM + 1]) value and presented as the MEAN ± the SEM (standard error of the mean). ANOVA analyses were conducted for multiple group comparisons. A student *t*-test was performed to determine the significance of the differences between the two groups. The results were visualized using the R package (version 4.2.1) and GraphPad software (version 9.1.0).

## Data Availability

The original contributions presented in the study are included in the article/**Supplementary Material**, further inquiries can be directed to the corresponding author/s.
