# Supplementary figures and images for: Aberrant expression of multiple γ-glutamyltransferases is associated with tumor progression and patient outcome in prostate cancers
Source: Front Oncol. 2025 Feb 28;15:1518636. doi: 10.3389/fonc.2025.1518636 (PMC11906340; doi:10.3389/fonc.2025.1518636)

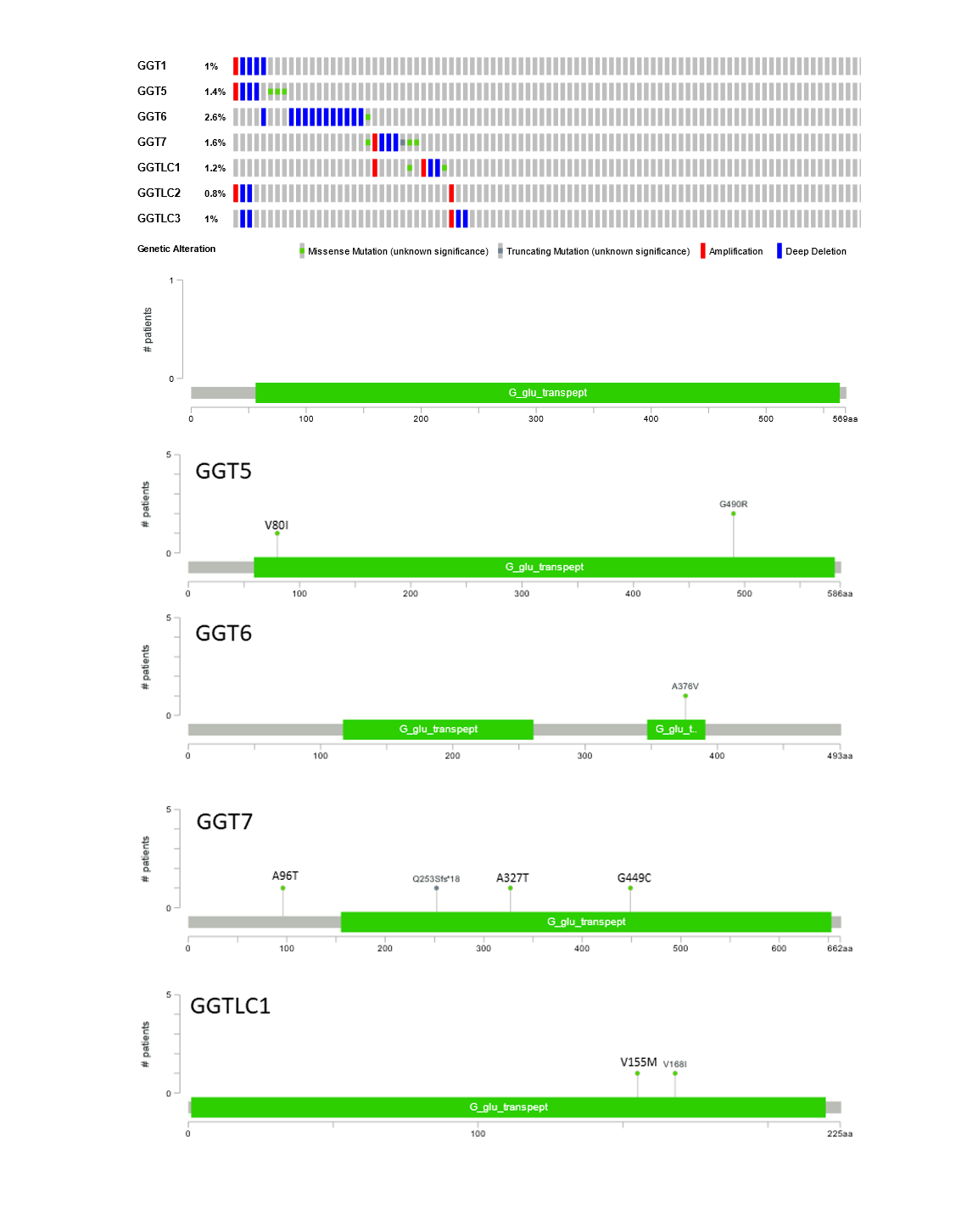

Supplement: Supplementary file 1 [file Image1.tif]

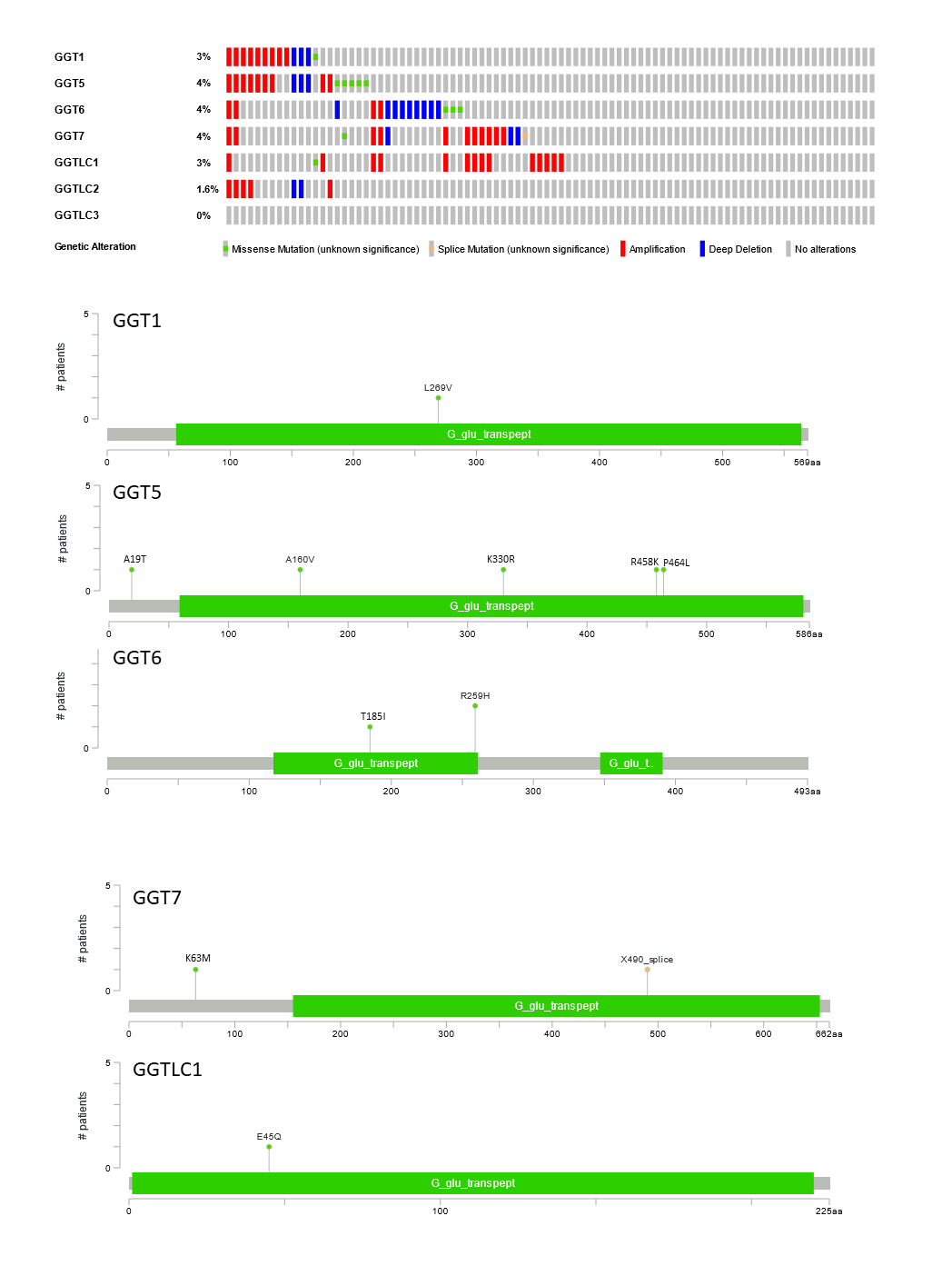

Supplement: Supplementary file 2 [file Image2.tif]
